# The effect of CSF drain on the optic nerve in idiopathic intracranial hypertension

**DOI:** 10.1186/s10194-019-1004-1

**Published:** 2019-05-23

**Authors:** Jan Hoffmann, Katharina Maria Kreutz, Christoph Csapó-Schmidt, Nils Becker, Hagen Kunte, Lucius Samo Fekonja, Anas Jadan, Edzard Wiener

**Affiliations:** 1 Department of Neurology, Charité – Universitätsmedizin Berlin, corporate member of Freie Universität Berlin, Humboldt-Universität zu Berlin, and Berlin Institute of Health, Charitéplatz 1, 10117 Berlin, Germany; 2 Basic and Clinical Neuroscience, Institute of Psychiatry, Psychology and Neuroscience, King’s College London, Wellcome Foundation Building, Denmark Hill Campus, London, SE5 9PJ UK; 3 Department of Neuroradiology, Charité – Universitätsmedizin Berlin, corporate member of Freie Universität Berlin, Humboldt-Universität zu Berlin, and Berlin Institute of Health, Charitéplatz 1, 10117 Berlin, Germany; 4 Faculty of Natural Sciences, Medical School Berlin, Berlin, Germany; 5 Department of Neurosurgery, Charité – Universitätsmedizin Berlin, corporate member of Freie Universität Berlin, Humboldt-Universität zu Berlin, and Berlin Institute of Health, Charitéplatz 1, 10117 Berlin Berlin, Germany

**Keywords:** Idiopathic intracranial hypertension, MRI, DTI, Optic nerve, Intracranial pressure, Lumbar puncture

## Abstract

**Background:**

Elevation of intracranial pressure in idiopathic intracranial hypertension induces an edema of the prelaminar section of the optic nerve (papilledema). Beside the commonly observed optic nerve sheath distention, information on a potential pathology of the retrolaminar section of the optic nerve and the short-term effect of normalization of intracranial pressure on these abnormalities remains scarce.

**Methods:**

In this exploratory study 8 patients diagnosed with idiopathic intracranial hypertension underwent a MRI scan (T2 mapping) as well as a diffusion tensor imaging analysis (fractional anisotropy and mean diffusivity). In addition, the clinical presentation of headache and its accompanying symptoms were assessed. Intracranial pressure was then normalized by lumbar puncture and the initial parameters (MRI and clinical features) were re-assessed within 26 h.

**Results:**

After normalization of CSF pressure, the morphometric MRI scans of the optic nerve and optic nerve sheath remained unchanged. In the diffusion tensor imaging, the fractional anisotropy value was reduced suggesting a tissue decompression of the optic nerve after lumbar puncture. In line with these finding, headache and most of the accompanying symptoms also improved or remitted within that short time frame.

**Conclusion:**

The findings support the hypothesis that the elevation of intracranial pressure induces a microstructural compression of the optic nerve impairing axoplasmic flow and thereby causing the prelaminar papilledema. The microstructural compression of the optic nerve as well as the clinical symptoms improve within hours of normalization of intracranial pressure.

## Introduction

Idiopathic intracranial hypertension (IIH) is defined as an elevation of intracranial pressure (ICP) without hydrocephalus or mass lesion and with normal CSF composition [1]. Currently the incidence is estimated at 0.03–2.36 cases per 100,000 of the general population [2–7] but it may be assumed that this figure will increase over the next decades as the disorder occurs mainly in obese women and the prevalence of obesity is increasing worldwide from year to year [8, 9]. The clinical picture of IIH is mainly characterized by a rather unspecific and highly variable headache [10] and visual deficits which result mainly from a pressure-induced papilledema [10–19]. While these symptoms are the main reasons for patients to seek medical advice, other symptoms such as cranial nerve palsies [20–24], olfactory disturbances [25–28] and cognitive impairment [29–32] may occur.

Visual alterations are observed in the vast majority of IIH patients [12, 33–35] but even so, the detailed mechanisms underlying this functional deficit remain far from being entirely understood. It may seem obvious that the elevation of ICP induces papilledema which in turn causes visual deterioration. However, there are many questions that remain unanswered. For example, it remains unclear if the pressure-induced changes affect exclusively the papilla of the optic nerve (ON) or if other regions along the visual pathway may be affected as well. It may be assumed that beyond the significant abnormalities observed in the papillar region, the ON suffers pressure-induced microstructural alterations in its myelinated retrobulbar segment [36] but it remains unclear if they are the direct consequence of elevated CSF pressure. In this context it is not known if and in which time frame these abnormalities would reverse upon a pressure relief obtained by a lumbar puncture. This aspect is particularly important as it is still unknown how long it takes to develop a papilledema after the increase of ICP. However, given its pathomechanism it is commonly accepted that the development does not occur immediately but requires a certain amount of time to develop upon an elevation of ICP [37, 38]. This delay suggests, that either an elevation of ICP does not immediately lead to an increase in pressure in the CSF space surrounding the ON or that additional mechanisms that go beyond the direct pressure-induced effects complement the functional changes that lead to visual deterioration. This hypothesis is supported by the observations that IIH-associated headache does not correlate to ICP [39, 40], papilledema [41] or any other IIH-related symptom [30] and that pressure relief does not induce a long-term remission of the head pain in the majority of patients [10, 42]. However, even in regard to the assumed short-term benefit of pressure-relief, objective data is limited. This is particularly the case for symptoms that may accompany IIH-related headache, such as nausea, photo- and phonophobia as well as tinnitus. These symptoms seem less significant at first, but in fact they may hamper the early diagnosis of IIH as they can lead the clinician to a misdiagnosis due to the clinical similarity to other headaches such as migraine [10, 11].

Quantitative diffusion tensor imaging (DTI) has been used to investigate various white matter diseases in vivo. It was shown that DTI is sensitive enough to detect axonal degeneration in rat glaucoma model [43] or the wallerian degeneration in acquired blindness [44] or to demonstrate pre- and post-chiasmatic diffusion tensor abnormalities in children with septo-optic dysplasia [45].

The aims of the present study were twofold. First, we sought to investigate if microstructural patterns within the ON may be affected by normalization of CSF pressure using state-of-the-art DTI magnetic resonance (MR) techniques. Secondly, we aimed at dissecting the characteristics of IIH-related headache and its accompanying symptoms as well as to analyze a potential improvement after the reduction of CSF opening pressure.

## Methods

The study was approved by the local ethics committee of the Charité - Universitätsmedizin Berlin (EA1/005/15). All patients participating in the study provided written informed consent. MRI measurements were performed between Feb. 11, 2015 and Nov. 25, 2015. Patients received two MRI scans during the study, one prior to the lumbar puncture and the second within 26 h (mean 15,54 h) after the lumbar puncture. In several of the cases the lumbar puncture in the study was not the diagnostic puncture, but all patients had no lumbar puncture within the last six months. Beyond the effect on microstructural alterations of the ON, changes of accompanying symptoms such as nausea, photo-, phono-, osmophobia and trigeminoautonomic symptoms were evaluated within that time frame.

### Patients

Eight patients of Caucasian descent (one male, seven female, mean age: 39,4 ± 3,7 years, BMI: 34,9 ± 1,5 kg/m^2^) diagnosed before with IIH according to the revised criteria established by Friedman et al. [1] were included in the study. Time of IIH diagnosis was within the last 18 months. Exclusion criteria included any medical conditions, IIH specific medications, surgical procedure that may affect ICP, significant weight loss or lumbar puncture within the last six months. IIH-associated headache was diagnosed according to the diagnostic criteria established by the International Headache Society (ICHD3 beta) [46].

### MR imaging (MRI)

MRI was performed on a 1.5 T scanner (Siemens Avanto Magnetom, Erlangen, Germany). In addition to the circularly polarized head coil, a loop surface coil (7 cm diameter) was placed and fixed over the most affected eye according to visual field defects based on the ophthalmological examination. For the evaluation of the ON and the optic nerve sheath (ONS) a coronal turbo spin echo (TSE) sequence was used (repetition time [TR] 6960 msec, echo time [TE] 99 msec, field of view [FOV] 85 × 85 mm^2^, matrix size of 256 × 256 [in plane resolution 0.332 × 0.332 mm^2^], slice thickness 2 mm and acquisition time [TA] 7 min 20 s). DTI images were acquired through a single-shot echo planar (EPI) sequence in axial orientation (TR 5448 msec, TE 88 msec, FOV of 192 × 192 mm^2^, matrix size of 128 × 128 [in plane resolution 1.5 × 1.5 mm^2^], slice thickness 3 mm, TA 11 min 44 s). The diffusion weighting (b value) was set to 0 and 1000 s mm^− 2^. Images were acquired with diffusion gradients in 126 directions with a number of acquisitions of 6, comprising 41 slices covering the whole brain. For the assessment of sinus vein stenoses (SVS) and the exclusion of sinus vein thrombosis (SVT) as secondary causes of intracranial hypertension, a 2D time-of-flight (2D TOF) venography (TR 23 msec, TE 6.5 msec, FOV 250 × 250 mm^2^, matrix size 512 × 512 [in plane resolution 0.488 × 0.488 mm^2^], slice thickness 2 mm, TA 7 min 40 s) was performed.

### Morphometric analysis

**Fig. 1:**
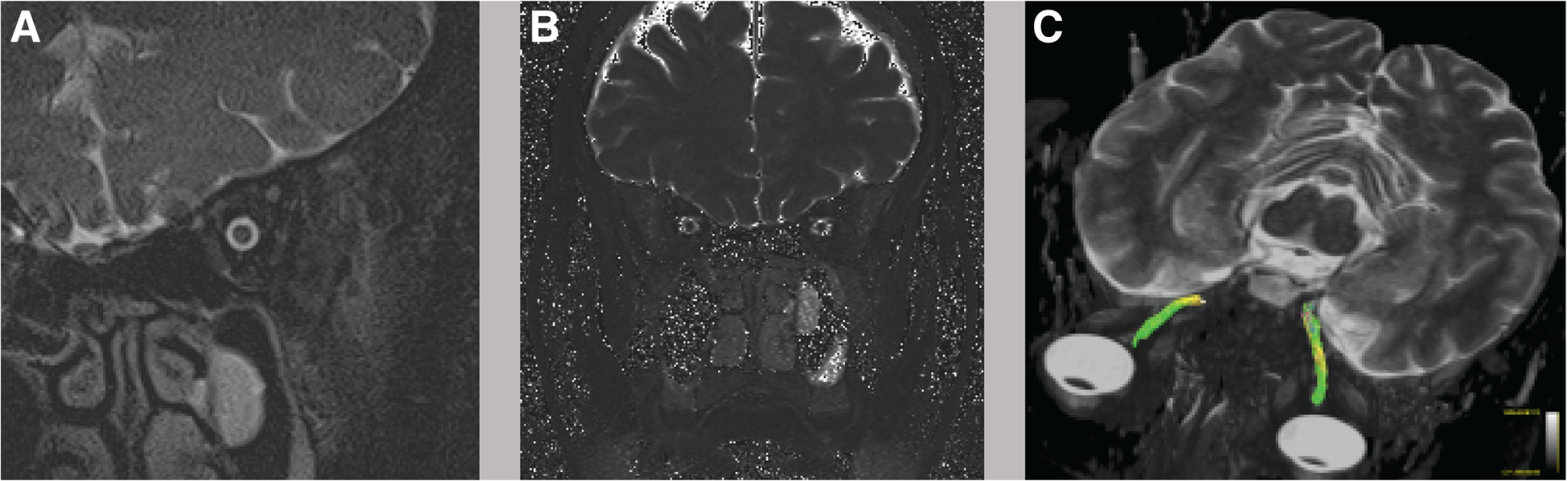
**a** shows an example of a coronal T2w image of the ON and ONS on which ON and ONS diameters where calculated. **b** shows the coronal T2-mapping. **c** illustrates the DTI measurements of the ON and depicts an MR-tractography of the ON

To allow a correlation between macroscopic findings and potential microscopic alterations, maximum ONS and ON diameters were measured in the most affected eye on coronal T2w images perpendicular to the ON in the slice with the maximum ONS diameter (Fig. 1a) measured with a MR microscopy surface coil. MRIs were evaluated by a neuroradiologist and a neurologist (E.W., J.H.) in a blinded fashion. To test the interrater agreement, we have calculated Crohnbach’s alpha for the ON diameter and for the FA values.

**Fig. 2:**
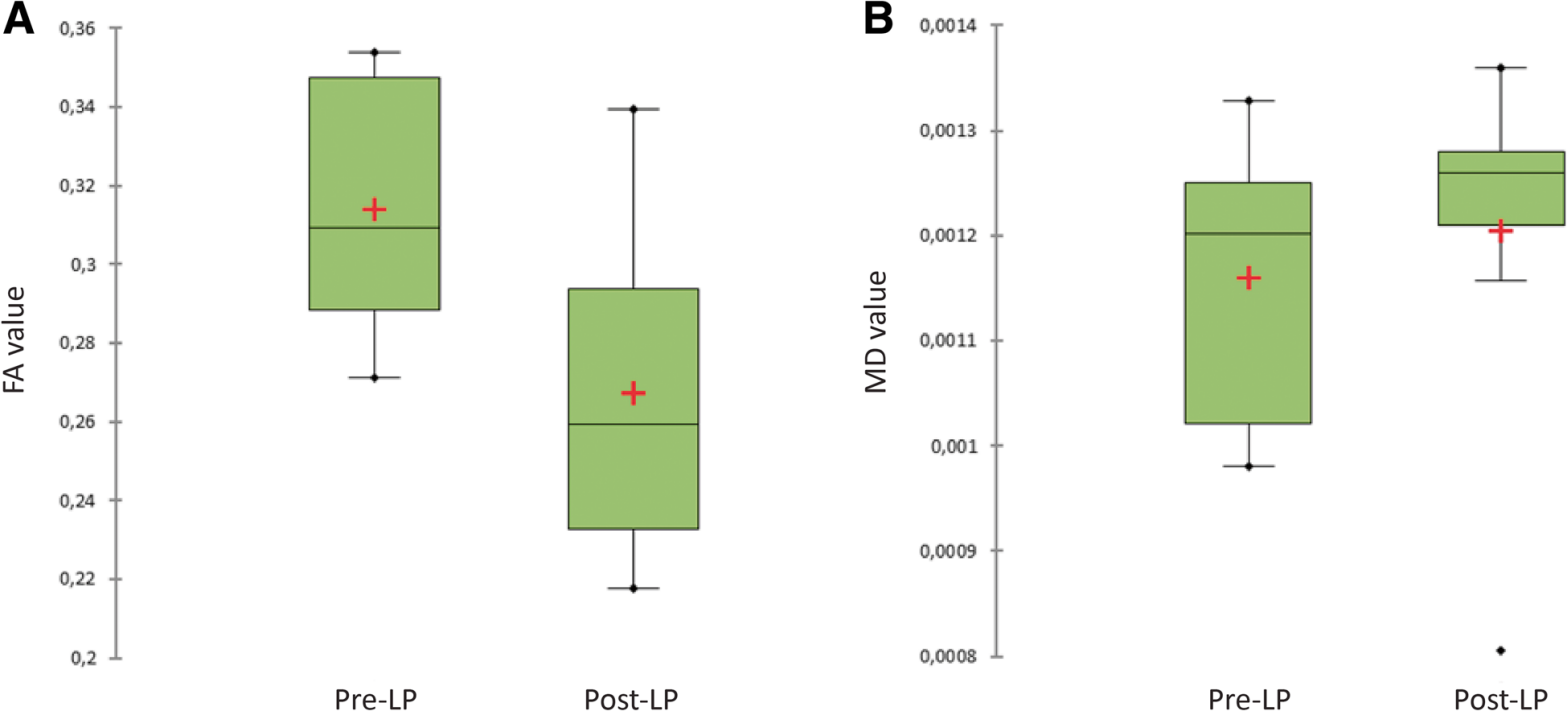
Illustrates the variation in the FA **a** and MD **b** values prior and after normalization of CSF pressure through lumbar puncture. The FA value was significantly reduced after normalization of CSF pressure (*p* = 0.038)

### MR-tractography

The MRI data has been denoised, corrected for eddy currents and motion and underwent a bias field correction for tractography purposes [47–49]. Probabilistic tractography has been performed with the iFOD2 algorithm and by using a seeding and an inclusion spherical region of interest (ROI) at the anterior and posterior positions of the optic nerves [50]. The iFOD2 is a probabilistic algorithm that takes Constrained Spherical Deconvolution (CSD)-estimated fiber orientation distribution (FOD) fields as input. Tracking parameters were set to default with a FOD amplitude cutoff value of 0.05, a streamline minimum length of 5 x voxelsize and a maximum streamline length of 100 x voxelsize.

The depicted image shows 5000 streamlines delineating a patients left and right optic nerves (Fig. 1c).

**Fig. 3:**
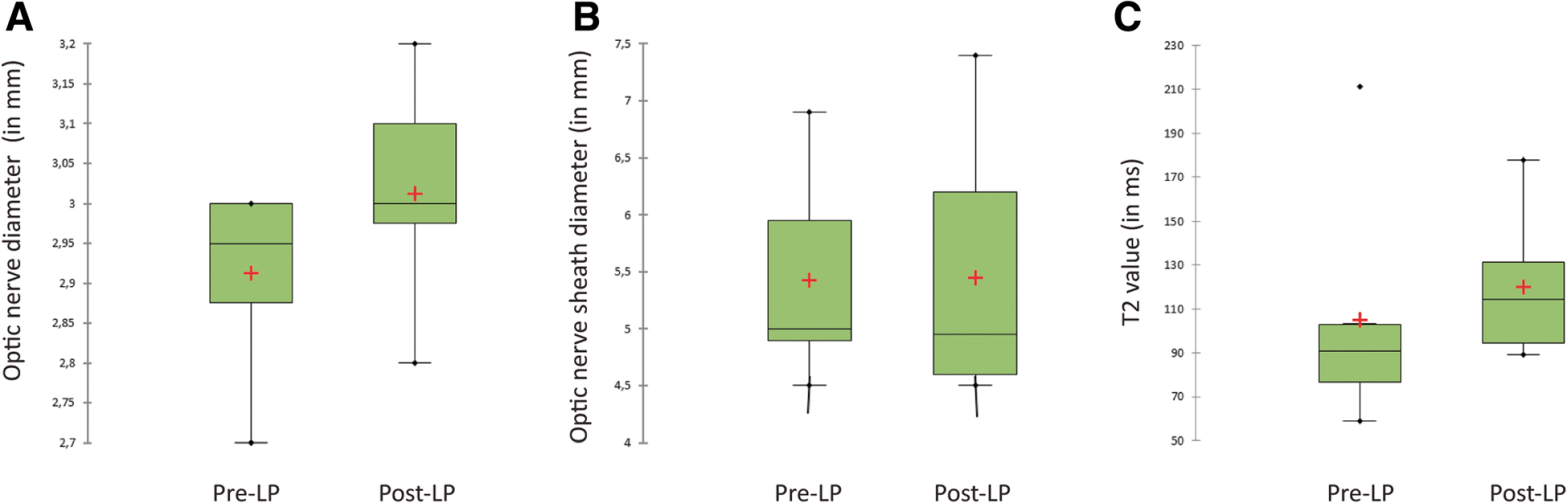
Illustrates the changes in ON **a** and ONS **b** diameters as well as in T2 values **c** before and after normalization of CSF pressure. The normalization of CSF pressure did not induce a significant change in any of the three parameters (ON *p* = 0.130, ONS *p* = 0.798, T2 *p* = 0.310)

### DTI analysis

DTI data were corrected for eddy current distortion and motion and underwent a bias field correction for tractography purposes [47–49]. DTI data were converted from DICOM into NIFTI format using the DICOM to NIFTI format converter dcm2nii (Dicom office toolkit DCMTK, https://dicom.offis.de/, Oldenburg, Germany). Voxelwise statistical analyses of fractional anisotropy (FA) and mean diffusivity (MD) data were performed using FSL 4.1.8 (FMRIB Software Library, Oxford, UK) [48, 51, 52]. The b0 data were used for brain segmentation using BET (Brain Extraction) [53]. For the segmentation of white and grey matter an automatic segmentation tool (FAST) was used. FA and MD values were calculated by reconstruction of diffusion tensors using FDT (FMRIB’s Diffusion Toolbox) and DTIFIT. Subjects’ FA and MD data were adjusted into an 1x1x1 mm standard space using tract-based spatial statistics (TBSS) [54] with FNIRT (FMRIB’s nonlinear registration tool), which uses a b-spline representation of the registration warp field [55]. For ROI analysis of the optic pathway, optic nerve and optic tract were outlined manually on MPRAGE and transferred to coregistered FA and MD images using Amira 5.3.2 (Visage Imaging Inc., San Diego, CA, USA) [56] (Fig. 1c).

**Fig. 4:**
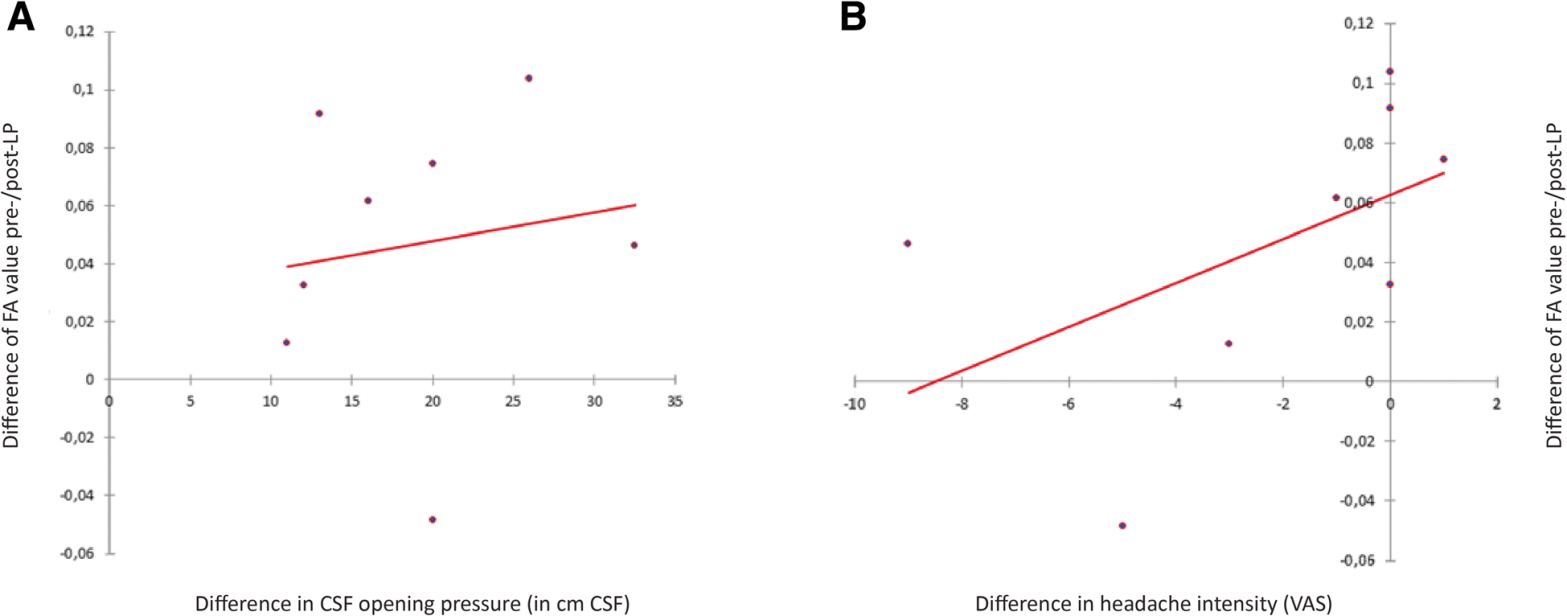
Depicts the correlations between the difference in FA value and CSF pressure **a** as well as between the difference of FA values and headache intensity **b** before and after normalization of CSF pressure. In both cases correlations were not significant (*p* > 0.05)

### T2 analysis

T2 mapping (TR 3100 ms, TE 13,8 ms - 165,6 ms with twelve TEs: 13,8 ms, 27,6 ms, 41,4 ms, 55,2 ms, 69 ms, 82,2 ms, 96,6 ms, 110,4 ms, 124,2 ms, 138 ms, 151,8 ms, 165,6 ms). T2 maps were online reconstructed by using a voxelwise, monoexponential nonnegative least-squares fit analysis (MapIt; Siemens, Erlangen, Germany) with a voxel size of 1.9 × 1.0 × 3 mm^3^. A ROI was manually delineated around the optic nerve on the T2 maps (Fig. 1b).

### Statistical analysis

Statistical analysis was performed using XLSTAT Version 2016.5 (Addinsoft SARL, New York, NY, USA). Normality of the data was calculated using the Shapiro-Wilk test. For the comparison of group means, the Mann-Whitney U-Test was used and data were expressed as median with 5th and 95th percentiles. Statistical significance was assumed at *p* < 0.05. To estimate the strength of the parameters Pearson’s correlation coefficient and correlation matrices were calculated. Crohnbach’s alpha was used to test for interrater variability.

## Results

### Morphometric analysis

The morphometric analysis revealed that CSF pressure reduction through lumbar puncture does not affect median ON and ONS diameters of IIH patients within the investigated time frame (ON *p* = 0.130, ONS *p* = 0.798) (Table 1, Fig. 1). Crohnbach’s alpha for the ON diameter was α = 0.72 which is accurate according to the small patient number.

**Table 1:** Summarizes the changes that normalization of CSF pressure induced on several morphometric and DTI measurements

	Pre-LP	Post-LP	Significance
CSF opening pressure [cm H_2_O]	28.50 [26.00; 43.00]	12.00 [7.00; 17.00]	*p* < 0.0001
FA	0.309 [0.271; 0.354]	0.259 [0.218; 0.339]	*p* = 0.038
MD	0.0012 [0.0001; 0.0013]	0.0013 [0.0001; 0.0014]	*p* = 0.382
ON diameter [mm]	2.95 [2.70; 3.00]	3.00 [2.80; 3.20]	*p* = 0.130
ONS diameter [mm]	5.00 [4.50; 6.90]	4.95 [4.50; 7.40]	*p* = 0.798
T2 [ms]	90.89 [59.00; 211.41]	114.44 [89.25; 177.81]	*p* = 0.310

Data are expressed as median with 5th and 95th percentile. Statistical analysis was performed using the non-parametric Mann-Whitney U-Test and significance was assumed at *p* < 0.05

### DTI analysis

Compared to the baseline measurement, FA values of the optic nerve were significantly reduced after lumbar puncture (*p* = 0.038) (Fig 2). In contrast, analysis of the MD values of the optic nerve remained unaffected by lumbar puncture (*p* = 0.382) (Table 1). Crohnbach’s alpha for the FA values was α = 0.48 which is acceptable according to the small patient number.

### T2 analysis

The comparison of the T2 value of the ON before and after lumbar puncture revealed no significant difference (*p* = 0.310) (Table 1, Fig. 3).

### Clinical assessment

The influence on clinical parameters was assessed in the same time frame. At the initial assessment seven out of eight patients (87,5%) presented with headache. Of those patients reporting headache, mean pain intensity, as assessed on a verbal analog scale ranging from zero to ten, was 2,94 ± 1,02. As outlined in Table 2, the majority of the patients had at least one accompanying symptom typically found in migraine, such as nausea, photo-, phono- and osmophobia, and slightly over a third of the patients had trigeminoautonomic symptoms such as lacrimation or rhinorrhea. With the exception of photophobia, which remitted in half of the patients after lumbar puncture, all the other accompanying symptoms including the trigeminoautonomic symptoms remitted completely within the observed time frame. The detailed description of accompanying symptoms as well as their development after lumbar puncture are listed in Table 2.

**Table 2:** Summarizes the characteristics of the study population as well as the characteristics of IIH-related headache and its accompanying features prior and after normalization of CSF pressure

Patient data	
Age [a]	39.4 y ± 3.70
Sex [M:F]	1:7
BMI [kg/m^2^]	34.90 ± 1.50
Volume CSF [ml]	23.38 ± 1.70
Clinical data	
Headache intensity before LP [x/10]	2.94 ± 1.02
Headache intensity after LP [x/10]	0.56 ± 0.32
Photophobia before LP [%]	50.00 (4/8 pat.)
Photophobia after LP [%]	25.00 (2/8 pat.)
Phonophobia before LP [%]	37.50 (3/8 pat.)
Phonophobia after LP [%]	0.00 (0/8 pat.)
Osmophobia before LP [%]	25.00 (2/8 pat.)
Osmophobia after LP [%]	0.00 (0/8 pat.)
Lacrimation before LP [%]	37.50 (3/8 pat.)
Lacrimation after LP [%]	0.00 (0/8 pat.)
Rhinorrhea before LP [%]	25.00 (2/8 pat.)
Rhinorrhea after LP [%]	0.00 (0/8 pat.)
Nausea before LP [%]	50.00 (4/8 pat.)
Nausea after LP [%]	0.00 (0/8 pat.)
Tinnitus before LP [%]	50.00 (4/8 pat.)
Tinnitus after LP [%]	0.00 (0/8 pat.)

Following the initial assessment and the first MRI scan all patients underwent a fluoroscopically guided lumbar puncture (E.W.) and CSF opening pressures were measured in the lateral decubitus position. The mean CSF pressure at baseline was 31,00 ± 2,20 cm CSF and was reduced to 12,19 ± 1,06 cm CSF. In the average 24 ml ± 4.8 ml of CSF were withdrawn. After lumbar puncture a second MRI scan and a reassessment of the clinical findings (headache and accompanying symptoms) was performed within 26 h. Following normalization of intracranial pressure headache severity improved in over 60% and photophobia in 50% of the patients. If nausea, phonophobia, osmophobia and/or trigeminoautonomic symptoms were present at baseline, these symptoms remitted completely in all of the affected patients within the observation period of 26 h (Table 2). In line with these findings, 50% of patients suffered from tinnitus at baseline and in all affected patients, symptoms remitted after lumbar puncture (Table 2).

### Correlation of imaging parameters with clinical parameters

The change of the headache intensity before and after lumbar puncture did not correlate with the change in the FA values of the optic nerve (*r* = − 0.626, *p* = 0.097) (Fig. 4) or with the change of the T2 values measured of the optic nerve (*r* = − 0.754; *p* = 0.084). Furthermore, no correlations were observed between the difference of CSF pressure before and after lumbar puncture with the change of the FA (*r* = 0.323; *p* = 0.435) (Fig. 4) and T2 values (*r* = − 0.314, *p* = 0.544) of the optic nerve.

## Discussion

In the present study we have shown in IIH patients that a reduction of ICP through a lumbar puncture does not induce a reduction of the ON and ONS diameters within the short time frame of 26 h. Furthermore, the DTI analysis of the ON revealed that the reduction in CSF pressure induced a decrease in the FA value reflecting a reduced restriction of the directionality of molecular diffusion. In contrast MD values remained unaffected after lumbar puncture as were the T2 values, indicating that the dysfunction of the ON is unlikely to involve an edematous process.

Neuroimaging techniques and in particular MRI are an essential component of the diagnostic workup of IIH to exclude secondary causes and to identify typical morphometric alterations commonly observed in IIH [1, 15, 18]. These alterations include the empty sella, a distension of the ONS (perioptic subarachnoid space) and a flattening of the posterior aspect of the optic globe [1, 39, 40]. The time lag between the initiation of the elevation of ICP and the establishment of these structural alterations remains unclear [37]. Perhaps even more important from a clinical perspective is the potential reversibility of these findings after normalization of ICP. However, if some or all of the abnormalities observed in the MRI improve after normalization of intracranial pressure is still debated. While an increasing number of publications report an improvement, in particular of the transverse sinus stenoses [57, 58], structured prospective studies addressing this question remain scarce and in regard to the ON and the ONS such studies, to the best of our knowledge, do not exist at all.

The present study investigated the IIH-related alterations of the ON and the ONS as well as their development after normalization of ICP using a loop surface coil on the orbita allowing the acquisition of high-resolution images of these structures.

The findings of our study indicate that the normalization of ICP does not have a short-term effect on morphological alterations of the ON. In contrast, the alteration of the FA value seen in the DTI suggests that elevated ICP affects microstructural integrity of the ON as the pressure-induced compression of the ON tissue leads to an increase in the directionality of molecular diffusion, a consequence that is reversed within hours of pressure relief. It may be speculated that the compression of the optic nerve as such and its microstructural consequences could add to the functional visual deficit induced by the papilledema, which is induced by a pressure-induced decrease in axoplasmic flow, but the answering of this question was beyond the scope of our study. The fact that we did not observe an alteration in the MD value of the ON supports the current pathophysiological concept that the pressure-induced edema is limited to the prelaminar part of the ON head [37, 59], which is located in front of the lamina cribrosa, and the adjacent retina [37], without affecting the postlaminar myelinated section of the ON [60].

Physiological axoplasmic flow depends on an intraaxonal pressure gradient between the ocular portion (maintained by intraocular pressure) and the extraocular portion (maintained by the pressure of the ON tissue) of the ON. The elevation of intracranial and ONS CSF pressure causes an increase of the retrolaminar tissue pressure that is reflected in the observations we made in the DTI of the ON. The elevated tissue pressure results in an imbalance of the intraaxonal pressure gradient that affects axoplasmic flow leading to an axoplasmic flow stasis in the ON head and a papilledema. Axoplasmic stasis leads to a swelling of the ON head and optic disc swelling that, if untreated, result in retinal hemorrhages and ischemia and finally to optic disc atrophy. This pathomechanism, the slow velocity of the axoplasmic flow and therefore the time needed to create an axoplasmic stasis and edema upon mechanical obstruction as well as the narrow capillary CSF communication in the optic canal explain the delay between the elevation of intracranial pressure and papilledema and why these changes are only observed in chronic but not in acute elevations of intracranial pressure [61].

Until now it remains to be clarified in which time frame the reduction of intracranial pressure reverses these findings. Studies investigating the effect on normalization of intracranial pressure on papilledema and visual disturbances generally investigate the evolution of these abnormalities in longer time frames. For example the NORDIC trial investigating the effect of acetazolamide assessed these parameters after 1 month [62]. To the best of our knowledge no study has investigated prospectively the short-term effect within hours of pressure normalization on the retrolaminar, myelinated section of the optic nerve using MR imaging techniques. However, previous studies on olfaction in IIH suggested, that the improvement and therefore the structural abnormalities, may improve within hours of normalization of ICP [28]. Our study shows that when tissue pressure on the ON is relieved, microstructural abnormalities are reversed but morphometric alterations remain unaffected within 26 h (mean 15:54 h) of normalization of ICP. While this seems obvious at first, it is surprising as the increase in CSF pressure within the ONS appears with a substantial delay compared to the intracranial CSF pressure and one would therefore suspect that upon pressure relief, the distention of the compressed optic nerve would occur much later.

Beyond the effect of normalization of ICP on microstructural alterations of the ON observed in the DTI, most of the patients presented accompanying symptoms commonly seen in migraine such as nausea, photo-, phono- and osmophobia and some had trigeminoautonomic symptoms. With the exception of photophobia, all the other accompanying symptoms remitted completely within the observed time frame. While the prevalences of these accompanying symptoms at initial presentation are similar to those observed by Yri et al. [10, 42], our study shows that most of these symptoms remit within hours of normalization of ICP. Interestingly Yri et al. observed that despite normalization of ICP headache and accompanying symptoms persist (or return) 1 month after pressure normalization [10, 11, 42]. Taken together, the findings suggest that pressure relief induces a short-lasting improvement of headache and accompanying symptoms which is not sustaining in a significant number of patients. The reasons for this rebound despite a normalization of ICP remain speculative but could be explained by the patients’ expectance of an improvement (placebo) as well as through a persistent sensitization of trigeminal neurons. In this case, while initially the elevated pressure may suffice to activate these sensitized neurons, this may not be the case shortly after the sudden fall in intracranial pressure but may return after the activation threshold of trigeminal neurons is adjusted to the new conditions. However, these hypotheses are highly speculative and the clarification of these mechanisms was beyond the scope of our study.

### Limitations

While the described findings are novel to the field, the study has a few but significant limitations that need to be taken into account when interpreting the data. First, the results of this study are based on 8 IIH patients so that they are of an exploratory nature and therefore need to be confirmed by larger studies. In this context the DTI results are only slightly significant given the limited amount of patients in this study. However, considering the exploratory nature of the study, the rather large error margin that affected the significance level and the fact that the decrease in the FA value after lumbar puncture reflects the finding of our previous study showing an increased FA value in IIH patients compared to healthy controls [36], suggest that the results are unlikely to change upon increase of the sample size. Furthermore, small or negative MRI findings in some patients on the second MRI scan could be explained with a faster recovery of intracranial CSF pressure after the lumbar puncture. Therefore, larger studies are clearly needed to confirm these findings.

Secondly, the lack of a control group is another limiting factor. However, as the study required a lumbar puncture and two MRI scans within a short time frame, a healthy control group was not included due to ethical considerations.

Thirdly, from a methodological perspective, the FA values provide a measurement that may be the sum of multiple structures and factors within a voxel. Therefore, FA values have to be interpreted in relation to anatomy, pathology and increase in intra- and extracellular water content. Because of the size differences between the axons and the voxels it is only possible and to observe optic nerve microstructure from a macroscopic point of view with DTI. Furthermore, a single voxel may be composed by fiber populations with different spatial orientation resulting in an average increase in FA, that is not due to changes in axonal structure. Therefore, FA is not always a reliable marker of white matter integrity and FA values have to be interpreted with caution, in particular in relation to clinical data [63, 64].

Fourthly, while papilledema was assessed externally by ophthalmoscopic examination at another campus or by the referring ophthalmologist to establish the diagnosis of IIH, we did not re-assess papilledema after lumbar puncture due to logistical reasons. We therefore had only the confirmation that patients had a papilledema but we did not have information on its extent. Therefore, we could not correlate the alteration of the optic nerve measured by changes of fractional anisotropy in relation to the papilledema. Given that this was intended as an exploratory study this question was beyond its scope. However, considering the pathomechanism of the papilledema induced by an elevation of ICP, it is highly unlikely that the papilledema may improve significantly in such a short time frame despite the differences measured in the optic nerve. In addition, assuming that the majority of patients had only a mild papilledema we would expect that our findings would have been more pronounced with more severe papilledemas or with higher CSF pressures.

Finally, in respect to the clinical outcome parameters, a more objective assessment of headache and its impact on quality of life would have been desirable. However, given the exploratory nature of the study and the focusing on the short-term effects post-LP we did not have a longer follow-up period that would have allowed us to use structured quality of life or disability questionnaires as these are commonly based on longer observational periods. However, in that context we would assume that the clinical improvement we observed post-LP is most likely of a short-lived nature as other groups have convincingly shown that most IIH patients do not have a long-term improvement of their headache even if opening pressure is normalized [65].

## Conclusion

The findings of our exploratory study show in a DTI analysis that the microstructural abnormalities, which elevation of ICP induces within the ON, can be reversed within 26 h of normalization of ICP. In contrast, high-resolution MRI revealed that morphometric alterations of the retrolaminar, myelinated section of the ON remain unaffected by ICP normalization. As alterations in the T2 values of the morphometric analysis as well as in the MD values of the DTI analysis were also not observed, we deduce that the affection of the retrolaminar section of the ON does not involve an edematous process. Therefore, the pressure-induced edema appears to be restricted to the prelaminar optic disc and retina.

The study further suggests, that alterations of the ON observed in the MRI as well as some clinical features of the disorder (headache and accompanying symptoms) may improve in the hours following normalization of intracranial pressure. Given the exploratory nature of this study, further studies are needed to confirm these findings.

## Key findings

### Before the study

- visual alterations are observed in the vast majority of IIH patients
- visual deficits result mainly from a pressure-induced papilledema
- the underlying mechanisms are only partially understood

### Study adds

- no obvious change in optic nerve T2 values after withdrawal of CSF
- small changes in fractional anisotropy of the optic nerve could be observed after lumbar puncture
- after withdrawal of CSF a small distention of the ON fibers occur

## Abbreviations

BMI: Body Mass Index
CSF: Cerebrospinal fluid
DTI: Diffusion tensor imaging
EPI: Single-shot echo planar
FA: Fractional anisotropy
FOD: Fiber orientation distribution
FOV: Field of view
ICHD: International Classification of Headache Disorders
ICP: Intracranial pressure
IHS: International Headache Society
IIH: Idiopathic intracranial hypertension
MD: Mean diffusivity
MRI: Magnetic resonance imaging
ON: Optic nerve
ONS: Optic nerve sheath
ROI: Region of interest
SVS: Sinus vein stenosis
SVT: Sinus vein thrombosis
TA: Acquisition time
TE: Echo time
TOF: Time of flight
TR: Repetition time
TSE: Turbo spin echo
